# Needle aspiration for treating iatrogenic pneumothorax after cardiac electronic device implantation: a pilot study

**DOI:** 10.1007/s10840-019-00596-x

**Published:** 2019-07-24

**Authors:** Dominika Domokos, Andras Szabo, Gyongyver Banhegyi, Balazs Polgar, Zsolt Bari, Peter Bogyi, Istvan Marczell, Leticia Papp, Robert Gabor Kiss, Gabor Zoltan Duray, Bela Merkely, Istvan Hizoh

**Affiliations:** 1 Heart and Vascular Center, Semmelweis University, Budapest, 1122 Hungary; 2 Department of Anesthesiology and Intensive Care, Semmelweis University, Budapest, Hungary; 3 Independent Researcher, Budapest, Hungary; 4 Department of Cardiology, Medical Center Hungarian Defense Forces, Budapest, Hungary

**Keywords:** Cardiac implantable electronic device, Complication, Pneumothorax, Chest tube drainage, Needle aspiration

## Abstract

**Purpose:**

Pneumothorax (PTX) following cardiac implantable electronic device procedures is traditionally treated with chest tube drainage (CTD). We hypothesized that, in a subset of patients, the less invasive needle aspiration (NA) may also be effective. We compared the strategy of primary NA with that of primary CTD in a single-center observational study.

**Methods:**

Of the 970 procedures with subclavian venous access between January 2016 and June 2018, 23 patients had PTX requiring intervention. Beginning with March 2017, the traditional primary CTD (9 cases) has been replaced by the “NA first” strategy (14 patients). Outcome measures were procedural success rate and duration of hospitalization evaluated both as time to event (log-rank test) and as a discrete variable (Wilcoxon-Mann-Whitney test).

**Results:**

Needle aspiration was successful in 8/14 (57.1%) of the cases (95% CI 28.9–82.3%), whereas PTX resolved in all patients after CTD was 9/9 (100%, 95% CI 66.4–100.0%, *p* = 0.0481). Regarding length of hospital stay, intention to treat time to event analysis showed no difference between the two approaches (*p* = 0.73). Also, the median difference was not statistically significant (− 2.0 days, *p* = 0.17). In contrast, per protocol evaluation revealed reduced risk of prolonged hospitalization for NA patients (*p* = 0.0025) with a median difference of − 4.0 days (*p* = 0.0012). Failure of NA did not result in a meaningful delay in discharge timing as median difference was 1.5 days (*p* = 0.28).

**Conclusions:**

Our data suggest that in a number of patients iatrogenic PTX may be successfully treated with NA resulting in shorter hospitalization without the risk of meaningful discharge delay in unsuccessful cases.

## Introduction

Iatrogenic pneumothorax (PTX) is a major complication of cardiac implantable electronic device (CIED) procedures. The incidence of PTX after CIED implantation varies between 0.7 and 1.7% according to recent publications [1–3]. It was found to be related to new subclavian venous access site and may be associated with low body mass index (BMI) and female gender [1, 4–6]. Pneumothorax requiring intervention is traditionally treated by large-bore chest tube drainage (CTD). Simple pneumothoraces, however, could also be treated successfully with the less invasive catheter aspiration as was shown by Delius et al. in 1989: for needle-induced PTX the success rate was 75% [7]. Similarly, needle aspiration (NA) has been found to be effective for primary and secondary spontaneous pneumothoraces in recent randomized trials [8, 9]. Compared with chest tube thoracostomy, needle aspiration is less traumatic and may help to shorten hospital stay thereby reducing costs. Though there is still no general consensus regarding this therapeutic modality, the British Thoracic Society recommended needle aspiration as a first-line therapy for primary spontaneous pneumothorax and as an option for small secondary spontaneous PTX as early as 2010 [10]. Yet, there are no data available about needle aspiration as a treatment option for iatrogenic PTX after CIED implantation. We hypothesized that, at least in a subset of patients, NA might also be effective in this clinical setting.

## Methods

### Study design, outcome measures

Using a prospective registry, we analyzed data of 970 consecutive patients who underwent CIED implantation, upgrade, or revision with at least one new subclavian vein access at the Medical Center Hungarian Defense Forces from January 2016 to June 2018. Of them, in 11 cases (1.1%), small pneumothoraces were resolved spontaneously, whereas 23 patients (2.4%) had intervention-requiring PTX. The decision about treatment was left to the operator’s judgment based on clinical symptoms and imaging by X-ray (for details, see Sect. 2.2). In cases necessitating intervention, traditional chest tube drainage was performed in all patients until March 2017 (“historic” control group, 9 cases), when the institutional protocol changed and primary CTD was replaced by the novel “needle aspiration first” strategy (14 patients). In cases with persistent PTX, thoracostomy has been done secondarily. We estimated procedural success rates and length of hospitalization as efficacy outcome measures. Safety of the procedures was assessed by the occurrence of infection, bleeding, and death of any cause during hospital stay. All four physicians involved in the interventions were high-volume operators. The study was conducted in accordance with the amended Declaration of Helsinki. The protocol was approved by the Institutional Ethics Committee on Human Research of the Medical Center Hungarian Defense Forces (approval number: 280-261/2018). All procedures were carried out with the patients’ written permission and all of them gave informed consent to participate in the registry.

### Needle aspiration

The institutional standard of care for large pneumothoraces was chest tube drainage with additional Heimlich valve under continuous suction. Since March 2017, with the change of the institutional protocol, needle aspiration was attempted for all PTXs requiring intervention regardless of the size of the PTX. The presence of PTX was checked using chest X-ray (CXR) in all patients. In case of clinical suspicion, CXR was done immediately; otherwise, it was performed the day after CIED implantation. Thus, PTX was detected within 24 h after the procedure. In addition to traditional interpleural distance measurements, size of the PTX was also estimated as percentage PTX volume according to Collins et al. [11]. The need for intervention was determined by clinical signs and symptoms and/or CXR findings according to the guideline of the British Thoracic Society: visible rim of more than 2 cm between the lung margin and the chest wall at the level of the hilum [10]. We implemented the checklist and technical protocol published by Pasquier in 2013 [12]. The puncture site was the second intercostal space in the midclavicular line a safe distance from both the generator, which is placed more laterally, and the internal mammary artery, which is located medial to the intended puncture track [13]. All patients received nasal oxygen therapy using a face mask. During the procedure oxygen saturation, heart rate, and blood pressure were monitored. The procedure was carried out under local anesthesia and aseptic conditions using a 16- or 18-gauge over-the-needle cannula, a three-way stopcock, and a 50-mL syringe. The volume of the aspiration was limited to 2500 mL since larger volumes indicate a persistent air leak requiring chest drain insertion [10]. In these failed needle aspiration cases, CTD was performed immediately. At the end of the intervention, the cannula was removed and a bandage was placed. Success of the procedure was checked by regular chest X-rays 1, 6, and 24 h following the intervention. The procedure was considered successful if the patient was asymptomatic and there was no residual PTX visible on the subsequent CXRs or it showed marked regression being at most a few millimeters at the apex of the lung. Patients with resolved PTX were discharged, whereas in persistent cases regular chest tube drainage was performed by a thoracic surgeon with continuous suction until no residual PTX was detectable on CXR. After removal of the chest tube, patients were observed for 24 h before discharge.

### Statistical analysis

Continuous baseline parameters were examined for normality with the D’Agostino-Pearson and Shapiro-Wilk tests. As none of the continuous variables showed normal distribution, the exact Wilcoxon-Mann-Whitney test was applied for comparisons. Variables in 2 × 2 contingency tables were assessed using Fisher’s exact test. We estimated procedural success rates and length of hospital stay as efficacy outcome measures. We analyzed raw data, since the small sample size did not allow the use of multivariable adjustment or propensity score–based techniques. Success rates of the different therapeutic approaches and their uncertainty were calculated using the exact binomial test, whereas Fisher’s exact test was performed for their comparison. Length of hospital stay was evaluated as “time to event” by Kaplan-Meier analysis and the exact log-rank test. In addition, it was analyzed as a discrete variable using the exact Wilcoxon-Mann-Whitney test. Besides intention to treat (ITT) and per protocol (PP) analyses, we also investigated the effect of secondary CTD (i.e., NA failure) on discharge timing. A two-tailed *p* value less than 0.05 was considered statistically significant. Computations were performed with R version 3.6.0 (R Foundation for Statistical Computing, Vienna, Austria) using the fBasics 3042.89, coin 1.30, rms 5.1-3.1, survival 2.44-1.1, survminer 0.4.4, and ggplot2 3.2.0 packages.

## Results

### Patient characteristics

Baseline demographic, clinical, and procedural characteristics of patients with intervention-requiring pneumothorax or no PTX and comparisons of the two treatment arms are summarized in Table 1. Patients with pneumothorax necessitating intervention had a lower body mass index and were more likely to be female. Demographic, clinical, and procedural characteristics of the two treatment groups were similar, yet coronary artery disease was more common among primary chest tube drainage patients. No safety outcome events (i.e., infection, bleeding, and death of any cause) occurred in any of the treatment arms during the studied period. Anatomically, the majority of the patients in the needle aspiration arm had partial PTX; there was only one massive PTX case with a totally collapsed lung (estimated median percentage PTX volume 39.5% (interquartile range (IQR) 32.75 to 54.75%)).

**Table 1:** Baseline demographic, clinical, and procedural characteristics

	Cardiac implantable electronic device procedures			Cases with pneumothorax requiring intervention		
Variable	No pneumothorax (*n* = 936)	Pneumothorax requiring intervention (*n* = 23)	*p* value	Primary chest tube drainage (*n* = 9)	Primary needle aspiration (*n* = 14)	*p* value
Age, median (IQR) (years)	74.0 (66.0–81.0)	77.0 (71.0–81.0)	0.25	78.0 (71.0–88.0)	76.5 (71.0–78.8)	0.68
Body mass index, median (IQR) (kg/m^2^)	27.3 (24.5–30.9)	24.4 (21.9–25.7)	< 0.0001	25.4 (24.4–26.0)	23.6 (21.7–25.3)	0.31
Female	330 (35.3%)	13 (56.5%)	0.0463	3 (33.3%)	10 (71.4%)	0.10
Hypertension	786 (84.8%)	18 (78.3%)	0.40	6 (66.7%)	12 (85.7%)	0.34
Diabetes mellitus	311 (33.2%)	5 (21.7%)	0.37	3 (33.3%)	2 (14.3%)	0.34
Chronic obstructive pulmonary disease	149 (15.9%)	6 (26.1%)	0.24	2 (22.2%)	4 (28.6%)	1.00
Coronary artery disease	299 (31.9%)	8 (34.8%)	0.82	6 (66.7%)	2 (14.3%)	0.02
Coronary artery bypass graft surgery	92 (9.8%)	2 (8.7%)	1.00	1 (11.1%)	1 (7.1%)	1.00
Ejection fraction ≤ 35%	354 (37.8%)	8 (34.8%)	0.83	4 (44.4%)	4 (28.6%)	0.66
Anamnestic malignancy	112 (12.0%)	4 (17.4%)	0.51	1 (11.1%)	3 (21.4%)	1.00
Anamnestic radiotherapy	28 (3.0%)	2 (8.7%)	0.16	0 (0.0%)	2 (14.3%)	0.50
Atrial fibrillation/flutter	344 (36.8%)	8 (34.8%)	1.00	3 (33.3%)	5 (35.7%)	1.00
Emergency device implantation	254 (27.1%)	5 (21.7%)	0.64	2 (22.2%)	3 (21.4%)	1.00
Multiple subclavian leads	459 (49.0%)	12 (52.2%)	0.83	4 (44.4%)	8 (57.1%)	0.68
Operator’s daily number of implantations, median (IQR)	3.0 (1.0–4.0)	2.0 (1.0–4.0)	0.46	2.0 (1.0–3.0)	2.0 (1.0–4.0)	0.87
Time at implantation, median (IQR) (h)	12.0 (10.0–15.0)	11.0 (8.5–14.5)	0.49	10.0 (8.0–14.0)	12.0 (9.3–15.5)	0.48

### Procedural success rates

**Fig. 1:**
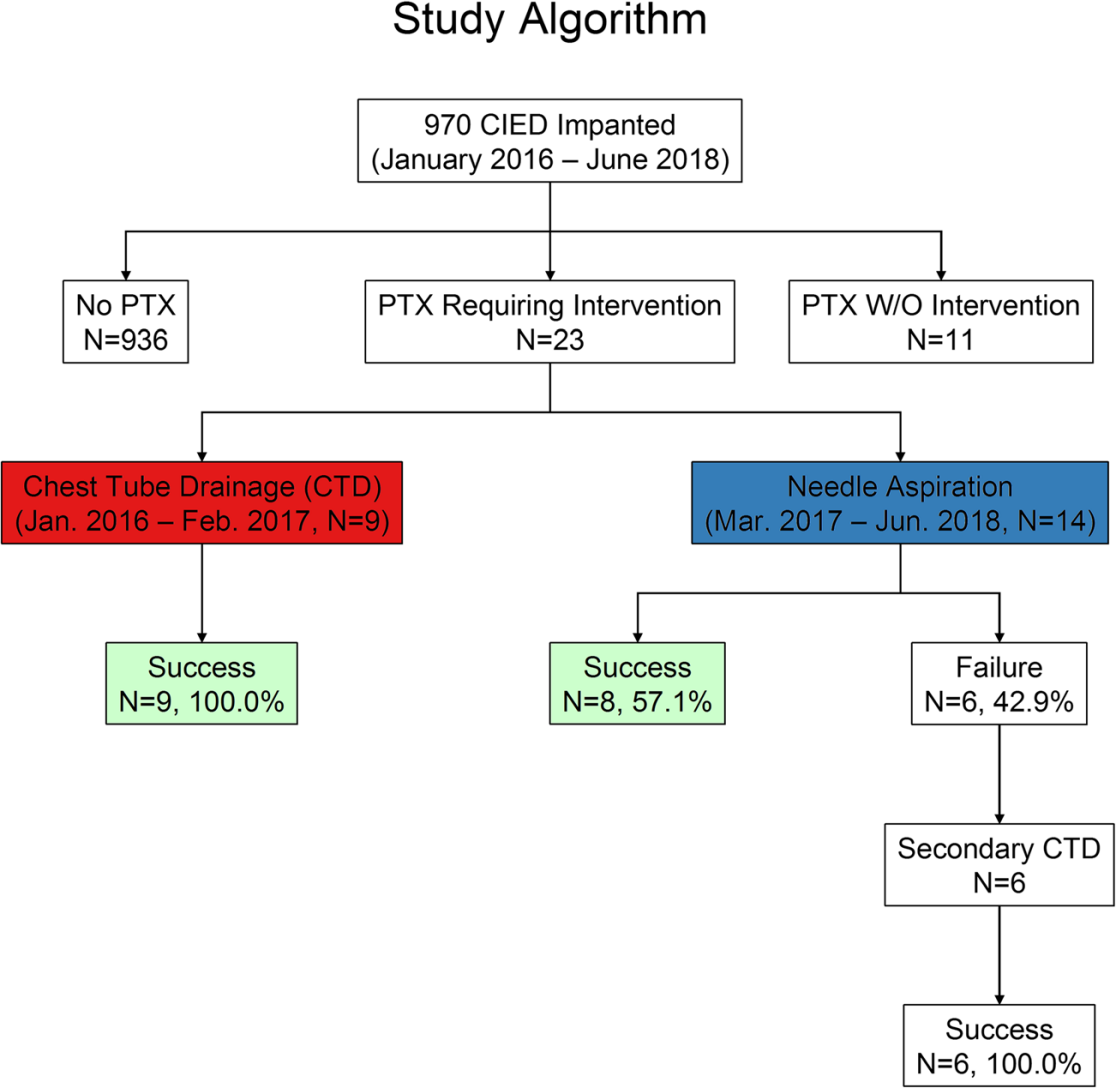
Study algorithm. Between January 2016 and June 2018, 970 patients underwent procedures with at least one new subclavian vein access. Of them, 23 patients had intervention-requiring pneumothorax (PTX) (for details, see text). CIED, cardiac implantable electronic device; CTD, chest tube drainage

Figure 1 shows the study algorithm. Out of 14 attempts, needle aspiration was successful in 8 patients (57.1%, 95% confidence interval (CI) 28.9 to 82.3%), whereas pneumothorax was resolved in all cases after chest tube drainage (9/9 (100%), 95% CI 66.4 to 100.0%, *p* = 0.0481). All patients with failed primary needle aspiration (6/14 (42.9%), 95% CI 17.7 to 71.1%) were treated successfully with secondary CTD.

### Length of hospital stay, intention to treat analysis

**Fig. 2:**
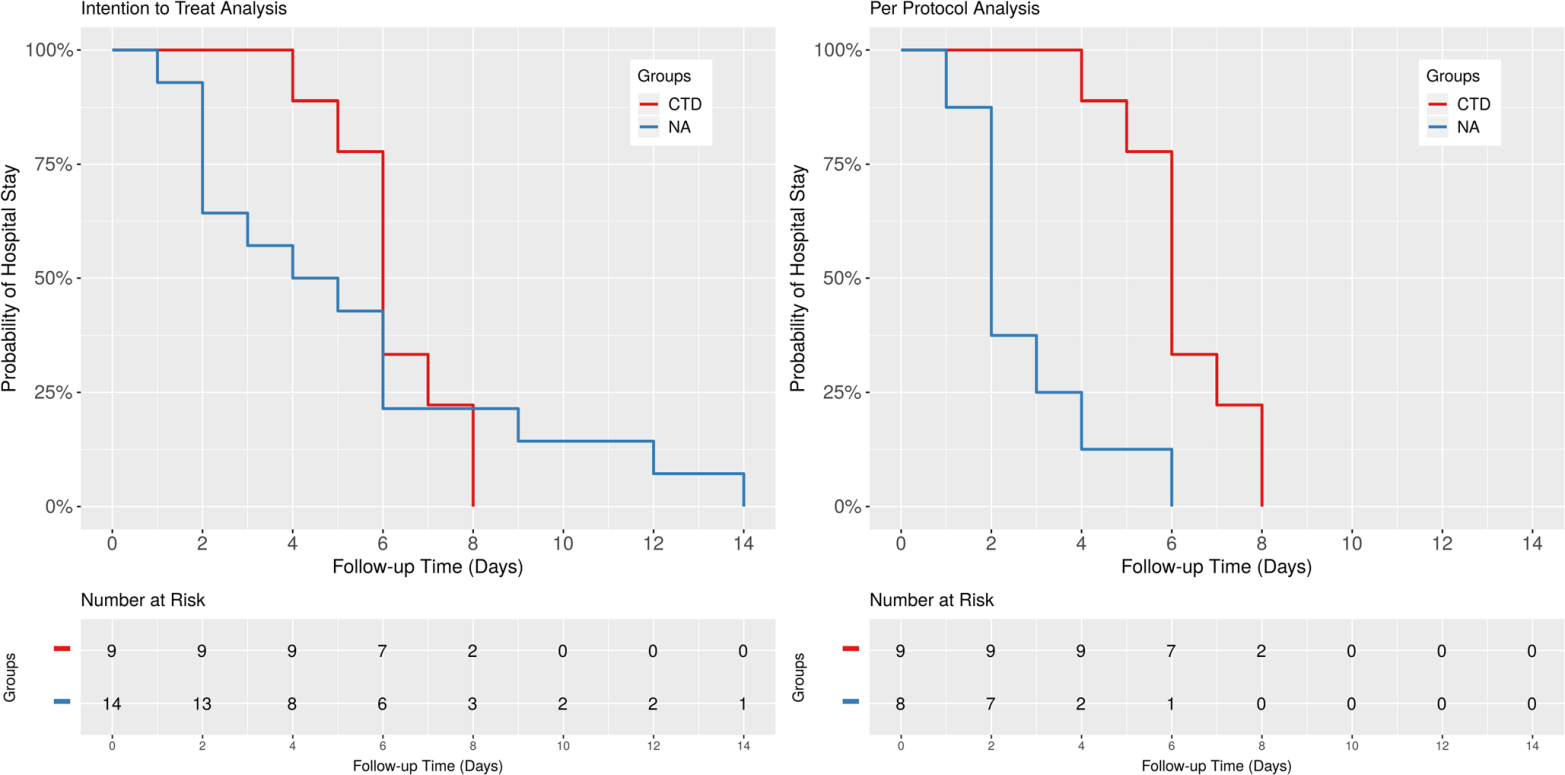
Length of hospital stay, Kaplan-Meier analysis. Left panel: Intention to treat analysis showed no statistically significant difference in time to event between the two treatment approaches according to the exact log-rank test (*p* = 0.73). Right panel: In contrast, per protocol evaluation revealed reduced risk of prolonged hospitalization for successful needle aspiration patients compared with primary chest tube thoracostomy cases (*p* = 0.0025; for details, see text). CTD indicates chest tube drainage, NA stands for needle aspiration

**Fig. 3:**
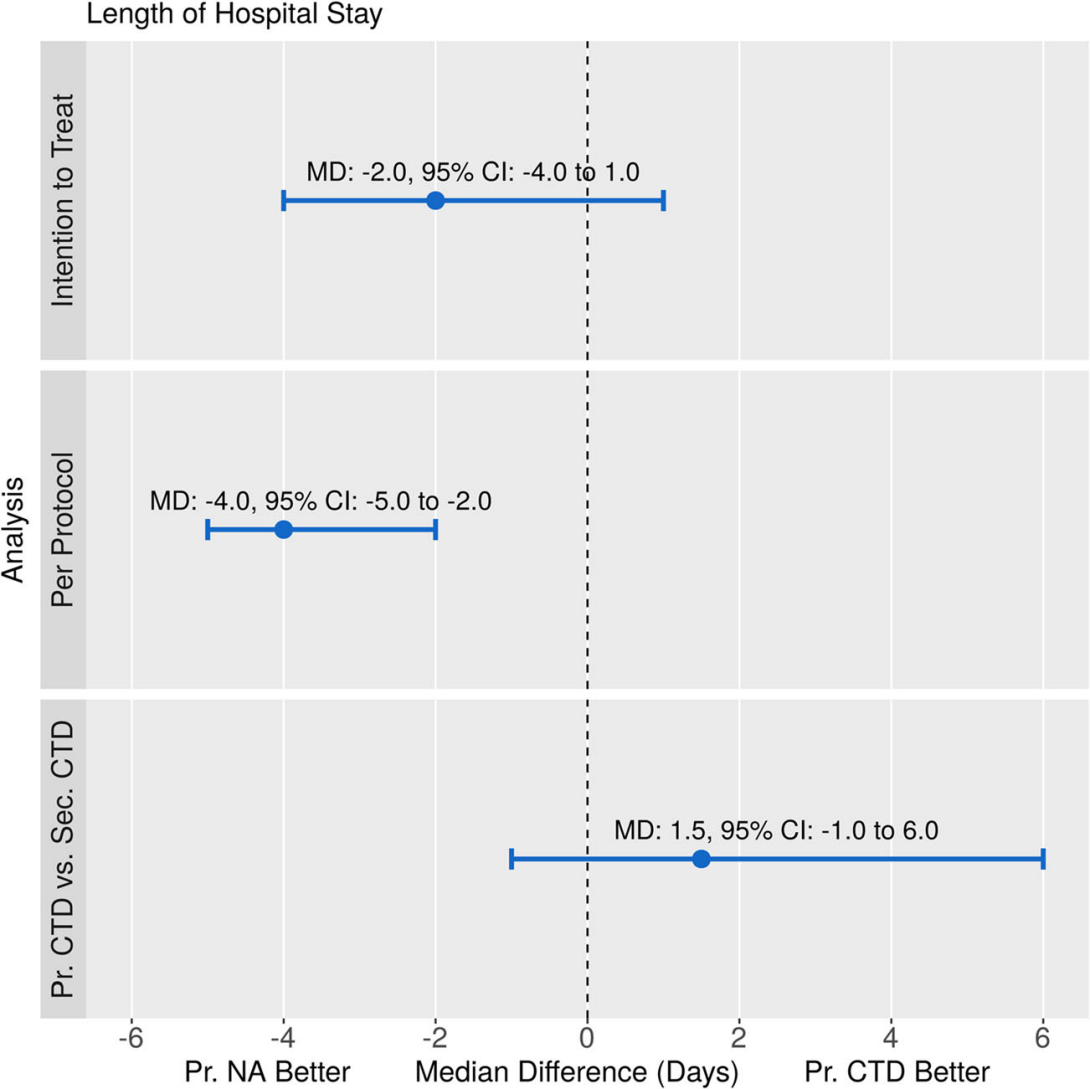
Length of hospital stay, median differences with 95% confidence intervals. Upper panel: Intention to treat analysis showed no statistically significant difference in median difference between the two treatment approaches according to the exact Wilcoxon-Mann-Whitney test (*p* = 0.17). Middle panel: In contrast, per protocol evaluation revealed shortened hospitalization for successful needle aspiration patients compared with primary chest tube drainage (*p* = 0.0012). Lower panel: Unsuccessful primary needle aspiration (i.e., secondary chest tube drainage) did not result in a statistically significant delay in discharge timing (*p* = 0.28). Pr., primary; Sec., secondary; CTD, chest tube drainage; NA, needle aspiration; MD, median difference

There was no statistically significant difference in time to event between the two treatment approaches according to the exact log-rank test (*p* = 0.73, Fig. 2, left panel). Similarly, median in hospital stays were comparable: 4.5 days (IQR 2.0 to 6.0 days) in the NA and 6.0 days (IQR 6.0 to 7.0 days) in the CTD group, median difference − 2.0 days, 95% CI − 4.0 to 1.0 days, *p* = 0.17, exact Wilcoxon-Mann-Whitney test (Fig. 3, upper panel).

### Length of hospital stay, per protocol analysis

In contrast, per protocol evaluation revealed reduced risk of prolonged hospitalization for successful needle aspiration patients (*n* = 8) compared with primary chest tube thoracostomy cases (*n* = 9, *p* = 0.0025, exact log-rank test, Fig. 2, right panel). Also, median length of hospital stays were different: 2.0 days (IQR 2.0 to 3.25 days) and 6.0 days (IQR 6.0 to 7.0 days), respectively, median difference − 4.0 days, 95% CI − 5.0 to − 2.0 days, *p* = 0.0012, exact Wilcoxon-Mann-Whitney test (Fig. 3, middle panel).

### Impact of failure of needle aspiration on discharge timing

Unsuccessful primary needle aspiration did not result in a statistically significant delay in discharge timing as the exact log-rank test *p* value was 0.10. Median duration of hospital stays were 7.5 days (IQR 6.0 to 11.25 days) for the failed NA (secondary CTD) and 6.0 days (IQR 6.0 to 7.0 days) for the primary CTD group, the median difference was 1.5 days, 95% CI − 1.0 to 6.0 days, *p* = 0.28, exact Wilcoxon-Mann-Whitney test (Fig. 3, lower panel).

## Discussion

### General considerations

Iatrogenic pneumothorax requiring intervention is a rare but serious complication of CIED procedures resulting in prolonged hospital stay. It is traditionally treated by large-bore chest tube drainage. Nevertheless, it has recently been shown that primary and secondary spontaneous pneumothoraces could also be treated successfully with the novel, less invasive needle aspiration technique [7–9, 12]. It is less traumatic for the patient and may help to shorten hospitalization. Despite the aforementioned data, there is no expert consensus regarding this therapeutic modality: The American College of Chest Physicians Delphi consensus does not advise needle aspiration, but rather supports primary chest tube insertion [14], while the British Thoracic Society guideline recommends needle aspiration as the first-line therapy for primary spontaneous pneumothorax and as an option for small secondary spontaneous pneumothorax [10]. Moreover, to the best of our knowledge, data about its possible role in the treatment of iatrogenic PTX after CIED procedures are completely missing. We hypothesized that, at least in a subset of patients, NA might also be effective in this clinical setting and conducted a preliminary single-center observational efficacy study using a prospective registry. We found that a subset of patients with iatrogenic pneumothorax following CIED procedures may successfully be treated with this technique resulting in shorter hospitalization without the risk of relevant discharge delay in failed cases.

### Risk of iatrogenic pneumothorax

The occurrence of PTX is associated with the puncture of the subclavian vein [1, 2]. Though the operators in this study used the cephalic vein cut-down technique whenever possible to avoid this complication, in a number of cases, puncture of the subclavian vein was inevitable. The occurrence of PTX in our cohort of patients with at least one new subclavian venous access was similar to previously reported data in this population, i.e., around 2.4% [2, 3]. It is of note, however, that the frequency of PTX may be further reduced using newer techniques like axillary vein cannulation [2] and ultrasound guidance [15]. We found, that PTX was more frequent among women and was associated with lower body mass index (Table 1). This is in accordance with previous findings of large registry-based studies [1, 4–6].

### Procedural success rate, length of hospital stay

In our cohort, the procedural success rate of NA was 57.1% (95% CI 28.9 to 82.3%). This is comparable with preceding reports on needle aspiration treatment of primary spontaneous pneumothorax with “immediate” success rates of 59.3 (95% CI 38.8 to 77.6%) to 66.7% (95% CI 48.2 to 82.0%) [16–18]. Yet, this rate was statistically significantly lower than that of the CTD arm (100%, 95% CI 66.4 to 100.0%, *p* = 0.0481). Regarding length of hospital stay, ITT analysis failed to reveal statistical difference between the two concepts (median difference − 2.0 days, 95% CI − 4.0 to 1.0 days, *p* = 0.17, Fig. 3, upper panel). Despite the statistically non-significant result, an effect size of this magnitude (i.e., a median decrease of 2.0 days) may be of clinical relevance, for which our preliminary study with limited sample size was not powered. Nevertheless, per protocol evaluation of the data shows that with primary NA, nearly 60% (57.1%) of the PTX cases could be discharged median 4 days earlier (Fig. 3, middle panel). On the other hand, discharge timing of the patients with failed needle aspiration (42.9%) was not delayed significantly (in both statistical and clinical sense): the median difference was 1.5 days (Fig. 3, lower panel) corresponding to the procedural protocol (with last control CXR after 24 h of observation, as described in Sect. 2) and the time needed for adjudication of success and actual discharge. Thus, a considerable number of patients with iatrogenic pneumothorax following cardiac implantable electronic device procedures may be successfully treated with the less invasive needle aspiration resulting in shorter hospitalization without the risk of meaningful discharge delay in unsuccessful cases. Moreover, these findings may raise the possibility that, despite the statistically non-significant results of the ITT analysis, the strategy of primary needle aspiration may have a clinical potential relevant effect on discharge timing. Of course, testing this hypothesis requires further studies with larger sample size.

### Technical considerations

Prior works pointed out the importance of the correlation between chest wall thickness and body mass index on proper selection of catheter length [13, 19]. The BMI of our patients was less than 31 kg/m^2^ and the pleural space could be reached in every patient with a regular 4.5-cm-long over-the-needle cannula. Also, the intended puncture track, i.e., second intercostal space in the midclavicular line, should be carefully located to minimize the risk of internal mammary artery injury [13].

### Limitations

Our results are based on observational data from a single institutional registry with inherent bias at treatment assignment. The limited sample size did not allow the use of multivariable adjustment or propensity score–based techniques to account for biased covariates. Also, we could not perform uni- and multivariable analysis of possible predictors of procedural success (e.g., size of the pneumothorax) because of lack of appropriate statistical power. Moreover, our pilot study was not powered to detect a clinical potential meaningful effect (i.e., a median decrease of 2.0 days) of primary needle aspiration on length of hospital stay in the intention to treat analysis. Furthermore, being primarily an efficacy analysis, it did not address potential adverse effects related to needle aspiration because of lack of power (no infection, bleeding, or death occurred in any of the treatment arms during the hospital stay). Also, we do not have follow-up efficacy and safety data. Therefore, future multicenter randomized studies or analysis of large national registries using statistical techniques that allow accounting for biased baseline covariates are warranted, particularly given the low incidence rate of this complication.

## Conclusion

Our preliminary data suggest that in a number of patients iatrogenic pneumothorax following cardiac implantable electronic device procedures may be successfully treated with needle aspiration resulting in shorter hospitalization without the risk of meaningful discharge delay in unsuccessful cases.
